# Case Report: Bezoar in allograft duodenum—rare cause of bowel obstruction 13 years post-pancreas transplant

**DOI:** 10.3389/frtra.2026.1706203

**Published:** 2026-02-02

**Authors:** Marlena Urvater, Cora Ianiro, David S. Bruce, Tuesday Jensen, Erik B. Finger, Justin Barr

**Affiliations:** 1 The University of Queensland—Ochsner Clinical School, New Orleans, LA, United States; 2 Department of General Surgery, Ochsner Clinic Foundation, New Orleans, LA, United States; 3 Department of Surgery, Division of Transplantation, University of Minnesota, Minneapolis, MN, United States

**Keywords:** bezoar, complication, pancreas transplant, pancreatic allograft, small bowel obstruction

## Abstract

Small bowel obstruction (SBO) following pancreatic transplantation is a recognized complication, most often caused by adhesive disease and hernias. To our knowledge, a pancreatic allograft duodenal bezoar has not been previously described as a cause of obstruction. A 69-year-old female presented to the emergency department (ED) with severe abdominal pain and computed tomography (CT) findings concerning for a SBO. Given imaging characteristics concerning for a mass and her complete obstruction, we performed an immediate exploratory laparotomy. Intraoperatively, a large, solidified bezoar within the pancreatic allograft duodenum and a smaller bezoar causing a SBO in the efferent jejunal limb were removed. This case reveals a previously undescribed cause of life-threatening obstruction within a pancreaticoduodenal allograft and highlights the importance of considering rare entities in pancreas transplant patients to preserve graft function and prevent further complications.

## Introduction

Small bowel obstruction (SBO) is a recognized complication following pancreas transplantation, most commonly resulting from adhesions or hernias (1). Less frequently, intussusception or posttransplant lymphoproliferative disease may be implicated (2, 3). To date, only one case of bezoar-related obstruction after a pancreatic allograft has been described, occurring 7 days postoperatively within the jejunojejunostomy of the Roux-en-Y loop (4). To the best of our knowledge, bezoar formation within a pancreatic allograft duodenum has not been previously described.

## Case description

A 69-year-old female presented to the ED with 12 h of excruciating, diffuse abdominal pain and non-bloody, non-bilious vomiting. The pain began as a vague abdominal discomfort that persisted and acutely worsened after dinner. She denied experiencing any diarrhea, constipation, or urinary symptoms.

She has a complex past medical history including bronchiectatic lung disease with recurrent pneumonias and *Mycobacterium avium* complex requiring hospitalization, chronic kidney disease stage IV, iron deficiency anemia, anemia of chronic disease, chronic gastropathy, slow-transit constipation requiring enemata, steroid-induced osteoporosis, and monoclonal gammopathy of undetermined significance. She had no history of psychiatric illness. Her surgical history includes a pancreas-alone transplant in 2012 for type 1 diabetes. The graft was placed head-down with systemic venous drainage, a Y-graft to the common iliac artery, and a distal duodenojejunostomy for enteric drainage. She underwent one reoperation on post-op day 11 for a fluid collection. She was initiated on triple immunosuppressive therapy (steroids, tacrolimus, mycophenolate), but neurological symptoms prompted a shift to cyclosporine. She had no other transplant-related complications and has remained insulin independent since surgery. In addition to her transplant, she has undergone multiple abdominal operations including appendectomy, cesarean section, hernia repair, and hysterectomy. She continues daily immunosuppression with prednisone and cyclosporine.

## Diagnostic assessment

**Figure 1:**
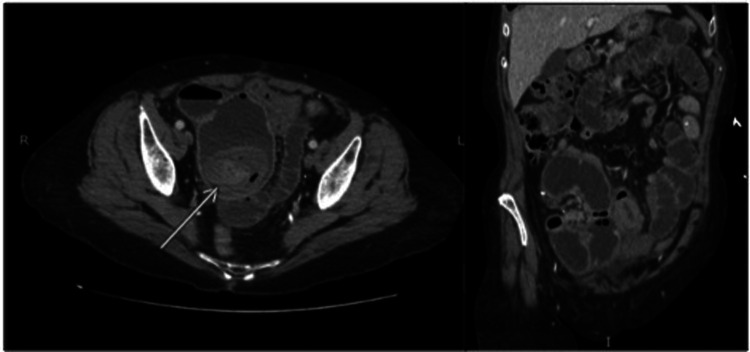
Preoperative abdominal computed tomography. Preoperative abdominal computed tomography demonstrating air fluid levels and dilated loops of small bowel. The findings were concerning for small bowel obstruction due to intussusception and localized perforation.

Upon examination, her temperature was 36.8°C, pulse rate was 85 bpm, and she was hypertensive with a blood pressure of 164/67 mmHg. She had severe, diffuse abdominal tenderness with rebound tenderness and guarding. Complete blood count demonstrated leukocytosis to 18.54 × 10^3^/μL. Hemoglobin and hematocrit were normal at 13.1 g/dL and 38.9%, respectively. The metabolic panel showed normal electrolytes with a slightly elevated from baseline creatinine of 1.8 mg/dL. Lipase was mildly elevated to 73 U/L. Glucose and calcium were normal, and HgbA1C was 5.0%. A CT scan of the chest, abdomen, and pelvis demonstrated dilated loops of small bowel in the lower abdomen. The radiologist reported intussuscepted small bowel in the pelvis with peripheral fluid and foci of gas concerning for contained perforation (Figure 1). There were no radiologic signs of pancreatitis.

The differential diagnoses included adhesive small bowel obstruction, hernia, intussusception, intraluminal malignancy, intraluminal hematoma, or gallstone ileus. Gallstone ileus was considered less likely due to the lack of pneumobilia, gallstones, or fistula seen on imaging. Given signs of peritonitis and CT evidence of a high-grade small intestinal obstruction in the setting of immunosuppression, the patient was taken emergently for an exploratory laparotomy.

## Therapeutic intervention

Her previous midline incision was opened, and an extensive lysis of adhesions was required. There were no signs of perforation. After a prolonged dissection, a hard mobile mass was identified in the efferent limb of the jejunum with dilated bowel proximally and decompressed bowel distally. The pancreas allograft was visualized, revealing a markedly dilated duodenal allograft with a much larger, freely mobile mass. The allograft appeared pink and well vascularized. Of note, there was no mesenteric lymphadenopathy or evidence of malignancy. The prior duodenojejunostomy was partially opened, and both masses were removed. The defect was closed in two layers.

## Follow-up and outcomes

**Figure 2:**
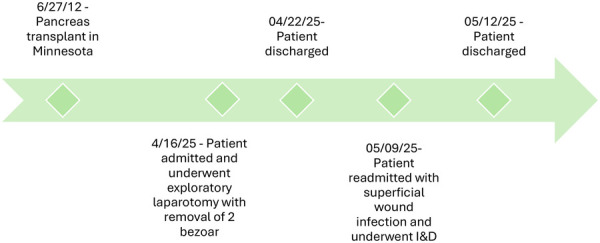
Schematic timeline summarizing the patients longitudinal clinical course.

The patient was transferred to the step-down unit, regained bowel function on postoperative day (POD) 4, and was discharged home on POD 6 with a functioning pancreas allograft. On POD 23, she was readmitted for a superficial wound infection that was treated with incision and drainage and antibiotics. She has since been discharged and is doing well (Figure 2).

**Figure 3:**
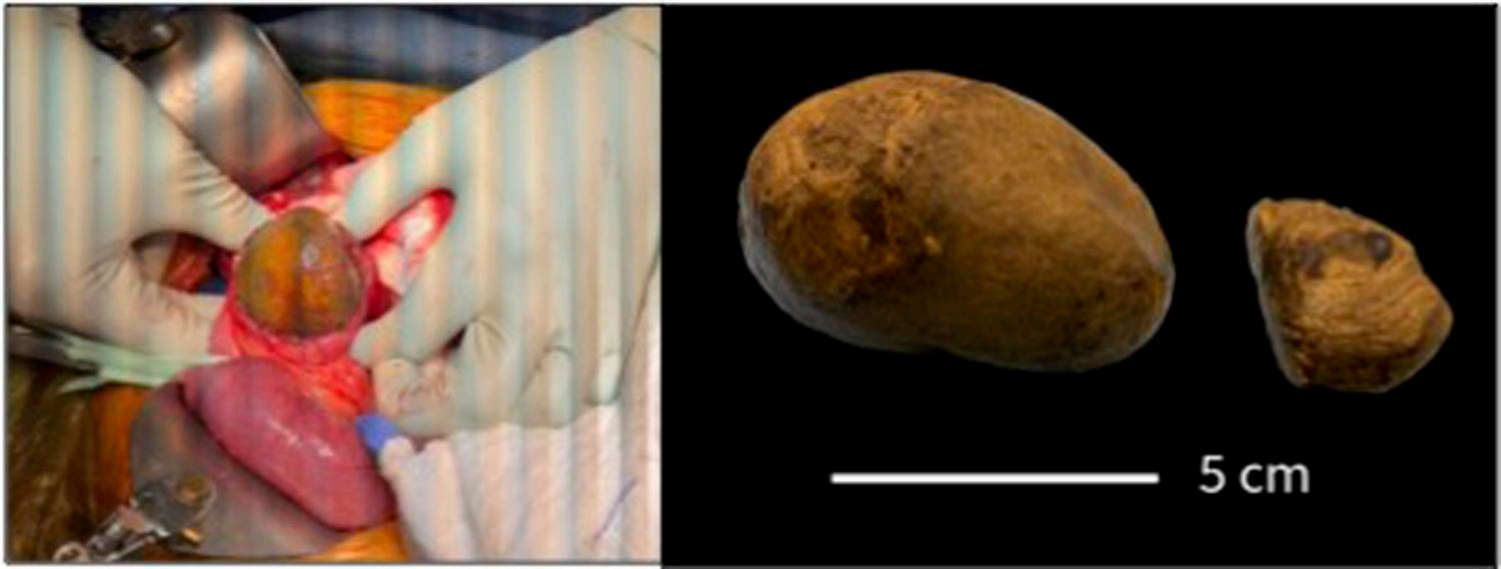
Intraoperative view of two bezoars. Two large, solidified masses were removed from the duodenal allograft of the transplanted pancreas.

Gross investigation revealed two large stones, measuring 7 cm × 5 cm × 4.5 cm and 3.5 cm × 1.5 cm × 3.5 cm, respectively. Pathology demonstrated two bezoars resembling solidified, impacted matter without a discernible major constituent such as hair, plants, or other foreign material (Figure 3).

## Discussion

Bezoars, which consist of indigestible materials such as hair (trico-bezoar), vegetable fibers (phyto-bezoar), or fruit residues, are an uncommon cause of SBO, accounting for 0.4%–4% of cases (5–7). Risk factors for bezoar include previous gastric surgery, gastroparesis or gastric outlet obstruction, hypothyroidism, trichophagia, certain medications, and poor mastication (8).

While rare, bezoars are most commonly found within the stomach (8). Bezoar formation within a pancreatic allograft duodenum leading to small bowel obstruction is an exceedingly uncommon phenomenon, and, to our knowledge, has not been previously reported in the literature. While there is a single case describing a bezoar obstructing the jejunojejunostomy of a pancreatic transplant, this occurred 7 days postoperatively, raising suspicion that the bezoar predated the transplant.

Pancreas transplants differ from the native pancreas primarily because of altered anatomy, drainage, and motility, which can create conditions not present physiologically. In the present case, the bezoar causing the obstruction likely formed within the donor duodenum and escaped into the recipient jejunum, while the other bezoar was too large to transit the anastomosis. The etiology of the bezoar formation remains unclear as pathologic examination was unable to determine the exact composition due to its solidified nature. Despite the unknown composition of the stones, several contributing factors may be considered. The patient's history of chronic slow-transit gastropathy raises the possibility of impaired gastrointestinal motility playing a role in bezoar development, although this alone does not fully explain stone formation inside the allograft duodenum. Moreover, many diabetic patients have motility issues without forming bezoars. The stasis of small bowel contents within the transplanted duodenal segment could have promoted bezoar formation, although why in this patient and not one of the thousands of other pancreas recipients remains unknown. The presence of two separate stones suggests some idiopathic combination of dietary, digestive, biochemical, and medication-related factors that predisposed her to this pathology. The mechanism may also be analogous to gallstone ileus, in which an intraluminal stone causes an obstruction at a point distal to its origin. Although the gross appearance was suggestive of a bezoar, definitive characterization could not occur due to the inability to perform a chemical analysis. It is possible this is not a true bezoar, but rather a fecaloma, cast, or another type of stone.

This case underscores the importance of considering bezoar as a rare but significant cause of small bowel obstruction in pancreas transplant recipients. Potential complications include not only bowel obstruction but also graft pancreatitis, anastomotic disruption, and graft loss, highlighting the importance of timely recognition and intervention (9). This case also highlights a previously unreported long-term complication of a pancreas transplant, bezoar formation in the pancreatic allograft duodenum with associated small bowel obstruction. While rare, bezoars should be considered in the differential for small bowel obstruction post-pancreas transplant. Early recognition and intervention are required to prevent further complications. This report adds to the literature regarding potential pancreas transplant complications and emphasizes the importance of considering atypical causes of small bowel obstruction in this unique population.

## Patient perspective

When I came into the hospital, I was in excruciating abdominal pain, and no one knew at first what was causing it. The doctors told me that even without a clear answer, I needed emergency surgery for an obstruction potentially involving my pancreas transplant. That was terrifying. For 12 years, my pancreas transplant had worked without a problem, and suddenly, I was faced with the possibility of losing it. The thought of going through surgery without knowing what was wrong was overwhelming. It seems as if the doctors were telling me that many horrible things could happen during the surgery. Right before I was taken to the OR, I said to my husband of 49 years that I did not think that I was going to make it out alive. I was so scared that I might lose everything. My husband, children, and my siblings were in a state of mental desperation not knowing what the doctors were going to find.

After surgery, I was unsure about what had happened, but once the doctors explained that they had found and removed two large stones, I felt both surprised and deeply relieved. It was reassuring to know that the cause of my pain had been identified and fixed, and most importantly, that my pancreas transplant had been saved. I was grateful not only for the outcome but also for finally understanding what had happened to me. The surgeon told my husband that in his many years of practice, he had never encountered anything like this. As of today, it is still a mystery what caused the stones.

## Informed consent

This project qualifies as a single-patient case report and does not meet the federal definition of human subjects research; therefore, an institutional review board (IRB) approval was not required. No prospective data collection or experimental intervention was performed. The case involves minimal risk and does not include any identifiable patient information. Written informed consent was obtained from the patient for publication of the clinical details.

## Data Availability

The original contributions presented in the study are included in the article/Supplementary Material; further inquiries can be directed to the corresponding author.
